# Pulmonary alveolar proteinosis induced by hydrofluoric acid exposure during fire extinguisher testing

**DOI:** 10.1186/s12995-015-0048-7

**Published:** 2015-02-25

**Authors:** YuJin Kim, JiYoung Shin, ShinMyung Kang, SunYoung Kyung, Jeong-Woong Park, SangPyo Lee, SangMin Lee, Sung Hwan Jeong

**Affiliations:** Division of pulmonary, Department of Internal Medicine, Gachon University Gil Medical Center, 1198 Guwol-dong, Incheon, Namdong-gu 405-760 ROK

**Keywords:** Hydrofluorocarbons (HF), Hydrofluoric acid (HFA), Pulmonary alveolar proteinosis (PAP)

## Abstract

**Introduction:**

Automatic fire suppression systems use hydrofluorocarbons (HF) to extinguish fires chemically. At high temperatures, HF can release hydrofluoric acid (HFA), a toxic, potentially lethal gas.

**Case report:**

A 52-year-old male visited our Pulmonary Division with dyspnea of 8-months duration. He had been working at a facility that manufactured fire extinguishers. Bronchoscopy was performed and a transbronchial lung biopsy was taken from the right lower lobe. After the patient was diagnosed with pulmonary alveolar proteinosis (PAP), whole-lung lavage was performed. In this case, fire extinguisher gas induced pulmonary alveolar proteinosis. This material should be used with care and investigated further.

**Discussion:**

HFA is corrosive and penetrates organic materials, including body tissues. Depending on the mode of exposure, skin ulceration, pulmonary injury, or even systemic shock can result. This report describes PAP that developed after chronic, repeated exposure to fire extinguisher spray. Hydrofluoric acid can induce pulmonary disorders such as PAP.

## Background

Hydrofluorocarbons (HF) are used as extinguishing agents [1]. The most frequently used HF is 1,1,1,2,3,3,3-hepatofluoropropane, which is generally non-toxic and stable [1]. However, it can decompose over time, when exposed to high temperature or under certain environmental conditions [1]. Hydrofluoric acid (HFA), an aqueous form of HF, is highly water-soluble and a weak acid [2]. Exposure to HFA might not cause symptoms initially. Over time, however, fluoride ions bind to intracellular calcium or magnesium, causing liquefactive tissue necrosis or systemic electrolyte disturbance [3,4]. Tracheobronchitis, pulmonary edema, pneumonia, bronchospasm, and acute respiratory failure syndrome have been reported after HFA exposure [1,4-6]. However, there are no reports of pulmonary alveolar proteinosis (PAP) secondary to HF extinguishing agents. We report the diagnosis and successful treatment of PAP that developed after fire extinguisher testing. PAP has been reported in workers exposed to aluminum dust, paint, sawdust, silica, synthetic plastic fumes, and indium-tin oxide. However, there are no reports of PAP after repeated fire extinguisher use.

## Case presentation

**Figure 1:**
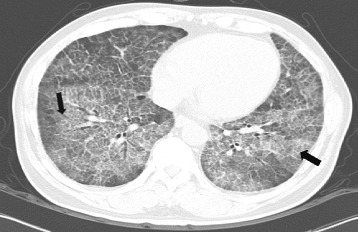
**Contrast enhanced computed tomography revealing Crazy paving patterned diffuse ground glass attenuation with inter/intralobular septal thickening, representing diffuse alveolar damage, both lungs (black arrow).**

**Figure 2:**
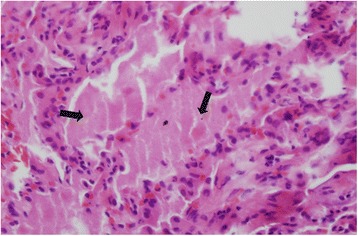
**Histology of the left lung, pulmonary alveolar proteinosis high magnification photomicrograph showing complete filling of alveoli with periodic-acid-Schiff-positive granular material in preserved alveolar architecture (black arrow) (PAS, x400).**

**Figure 3:**
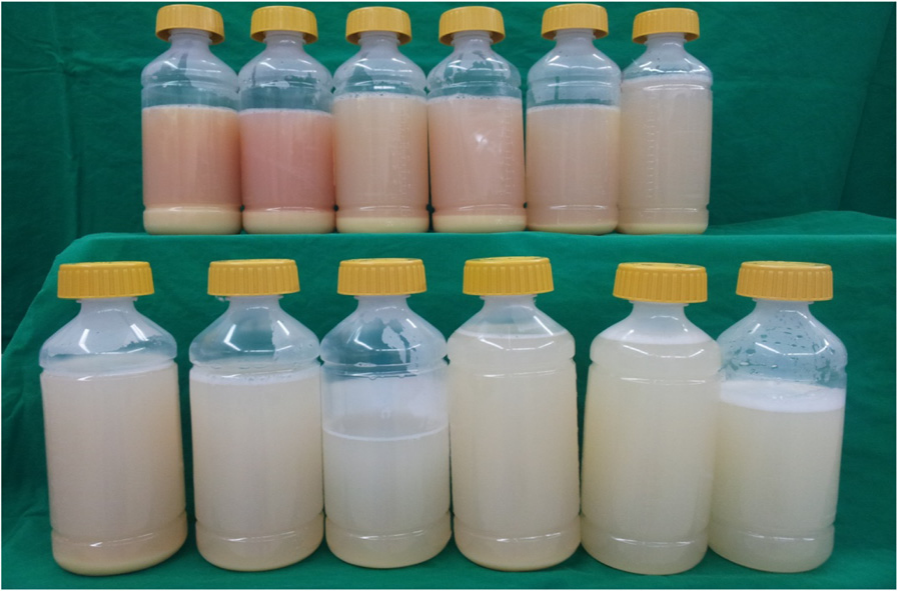
**Whole lung lavage fluid, first bottle (left and upper) is milky, opaque fluid and thick sediment layer.** Last bottle (right lower) is fade compared to previously bottle and no sediment.

A 52-year-old male visited our Pulmonary Division with dyspnea of 8-months duration. He had smoked for 30 years and had a 5-year history of hypertension for which he was prescribed medication. For 20 years, he had a desk job at a facility that manufactured fire extinguishers. Beginning 8 months ago, he was exposed to gas from fire extinguishers repeatedly, for less than 10 s each time. He complained of an intermittent cough that was relieved within a few hours. Two months before visiting our hospital, he was exposed to the gas over an extended period of time. The severity of his symptoms increased rapidly. His symptoms included dyspnea with a cough on exertion and eye irritation. He was exposed to the fire extinguisher spray roughly three times per week. On physical examination, he looked ill. His blood pressure and body temperature were normal. His lips were cyanotic. Rales were heard in both lower lungs. The initial oxygen saturation on room air was 88%. Arterial blood gas analysis showed a pH of 7.469, a PCO_2_ of 29.3 mmHg, PO_2_ of 79.7 mmHg, HCO_3_ 21.4 mEq/L, and saturation of 96.6% with a nasal cannula receiving supplemental oxygen at 3 L/min. Laboratory tests including blood and sputum were normal. Pulmonary function tests showed FEV_1_ 3.14 L (96%), FVC 3.45 L (77%), FEV_1_/FVC 91%, and DL_CO_ 42%. A simple chest x-ray revealed a bilateral pulmonary infiltration, while contrast-enhanced chest computed tomography showed ground glass opacities with a crazy paving appearance (Figure 1). Bronchoscopy with bronchial alveolar lavage (BAL) and a transbronchial lung biopsy of the right lower lobe were performed. Bronchoscopy showed proteinaceous secretions in both bronchial trees. The BAL fluid was milky and opaque. Microscopically, the transbronchial lung biopsy showed complete filling of alveoli with periodic-acid-Schiff-positive granular material in the preserved alveolar architecture (Figure 2). The patient was diagnosed with pulmonary alveolar proteinosis. Whole-lung lavage was performed under general anesthesia (Figure 3). On discharge, a simple chest x-ray showed improved lung haziness bilaterally. His respiratory symptoms improved. Sixteen months later, pulmonary function tests showed FEV_1_ 3.57 L (115%), FVC 4.34 L (104%), FEV_1_/FVC 82%, and DL_CO_ 81%.

### Discussion

Pulmonary alveolar proteinosis is a rare pulmonary disorder in which surfactant proteins and lipids accumulate within the intra-alveolar spaces [7,8]. PAP is classified into primary and secondary forms: secondary PAP is caused by hematological agents, infection, or the inhalation of dust or fumes [7-9]. PAP has been reported in workers exposed to aluminum dust, paint, sawdust, silica, synthetic plastic fumes, and indium-tin oxide [8]. However, there are no reports on PAP after fire extinguisher use, as occurred in our patient. The hydrocarbon used in fire extinguishers is 1,1,1,2,3,3,3-hepatofluoropropane, which is non-toxic under stable conditions, but can decompose into HF at high temperatures, particularly in combination with extended exposure to humidity, contaminants, certain metals, metal surfaces, or contact with certain liquids or vapors [1]. HFA is corrosive and penetrates organic materials, including body tissues. Depending on the mode of exposure, skin ulceration, pulmonary injury, or even systemic shock can result [2-5]. Wu *et al*. investigated HFA exposures occurring from 1991 to 2010: dermal exposure was the most frequent (83.6%), but inhalation only (7.1%), combined dermal and inhalation (1.9%), and combined ocular and inhalation (0.3%) exposure were also reported [4]. Kono *et al*. and Kawaura *et al*. also reported pulmonary injury accompanied by acute respiratory distress syndrome after HFA inhalation [5-7]. Zierold *et al*. reported that three US military personnel died of acute respiratory failure after using a fire extinguisher in their vehicle following a rocket-propelled grenade attack. They presumably inhaled the HFA generated from HF by the high temperature within the vehicle [1].

In our case, a high-temperature garbage incinerator was located in the same room where the patient performed the fire extinguisher spray test. Most likely, the HF was converted to HFA, which was then inhaled. Notably, we did not measure the concentration of autoantibodies to granulocyte–macrophage colony-stimulating factor (GM-CSF) in this patient. However, Cummings *et al*. asserted that inhalational exposure cannot be excluded when primary PAP is suspected [10,11]. In addition, Inoue *et al*. reported a dust inhalation history in 23% of primary PAP cases [11,12]. In our case, we surmise that the GM-CSF autoantibody status is less important. The limitation of this paper is a single case. Therefore we needed further studies to determine if there is an association with HFA.

## Conclusions

In conclusion, this report describing PAP that developed after chronic, repeated fire extinguisher spray exposure reveals that hydrofluoric acid can induce pulmonary disorders such as PAP in addition to pneumonitis and acute respiratory failure. These findings highlight the need for caution and greater attention to this risk.

## Consent

Written informed consent was obtained from the patient for publication of this case report. A copy of the written consent is available for review by the Editor–in–chief of this journal.
